# Amino acids and acylcarnitine production by* Chlorella vulgaris* and* Chlorella sorokiniana* microalgae from wastewater culture

**DOI:** 10.7717/peerj.7977

**Published:** 2019-12-03

**Authors:** Juan M. Ballesteros-Torres, Luis Samaniego-Moreno, Ricardo Gomez-Flores, Reyes S. Tamez-Guerra, Cristina Rodríguez-Padilla, Patricia Tamez-Guerra

**Affiliations:** 1 Facultad de Ciencias Biologicas (FCB), Departamento de Microbiología e Inmunología, Ave. Universidad s/n, Cd. Universitaria, Universidad Autónoma de Nuevo León (UANL), San Nicolás de los Garza, Nuevo León, México; 2 Departamento de Riego y Drenaje, Laboratorio de Calidad de Aguas, Universidad Autónoma Agraria “Antonio Narro” (UAAAN), Saltillo, Coahuila, México

**Keywords:** *Chlorella vulgaris*, *Chlorella sorokiniana*, Metabolomics, Growth conditions, Acylcarnitines, Amino acids

## Abstract

**Background:**

Microalgae are a widely distributed group of prokaryotic and eukaryotic photosynthetic microorganisms that use a number of substances present in wastewater to produce a variety of biotechnological and nutritional biomolecules.

**Methods:**

Production of****amino acids and acylcarnitine by *Chlorella vulgaris* and* Chlorella sorokiniana* was determined after 13 d of culture in wastewater, under various culture conditions. Wastewater was collected from “La Encantada” stream, located in Saltillo, Coahuila, Mexico. Microalgae was cultured at 23°C and natural day light, including the use of the following conditions: (1) extra light (12:12 light:dark cycles, 1,380 lumens), (2) agitation (130 rpm), and (3) both conditions, until exponential phase. Supernatant products were then analyzed by liquid chromatograph coupled to mass spectrometry. In addition, metabolomic profiles related to growing conditions were evaluated.

**Results:**

Amino acids and acylcarnitine production by *C. sorokiniana* and* C. vulgaris* resulted in higher Ala and Leu concentrations by *C. vulgaris* compared with control, where control produced Gly and Pro in higher amounts compared with *C. sorokiniana*. Tyr, Phe, Val, and Cit were detected in lower amounts under light and shaking culture conditions. High concentrations of C0 acylcarnitines were produced by both microalgae compared with control, where *C. sorokiniana* production was independent of culture conditions, whereas *C. vulgaris* one was stimulated by shaking. C4 production was higher by *C. sorokiniana* compared with control. Furthermore, C4, C6DC, C14:1, C14:2, and C18:1OH production by microalga was low in all culture conditions.

**Conclusion:**

Microalgae produced essential amino acids and nutritionally important carnitines from wastewater. In addition, *C. sorokiniana* biomass has higher potential as animal nutrient supplement, as compared with that of *C. vulgaris*.

## Introduction

Microalgae are a group of microorganisms that benefit from light and inorganic substances to produce organic molecules, useful for their metabolism, cell development, and growth (Bellou et al., 2014). They have been reported to serve as biotechnological tools for wastewater bioremediation, providing photosynthetic oxygen used by heterotrophs to oxidize organic matter (De Godos et al., 2010). Their potential to grow and reduce contaminants in fresh water has prompted their use for wastewater treatment (Ummalyma, Sahoo & Pandey, 2019), and supported evaluation of microalgae growth and production of secondary metabolites upon selected growing conditions, which may impact their biomass (Hu, 2004; Jin & Melis, 2003; Markou & Nerantzis, 2013). In addition, these microorganisms have been shown to produce proteins and fatty acids of nutritional value for human and animal consumption (e.g., dietetic supplements and food ingredients) and perform photosynthetic CO_2_ fixation with reduction of the greenhouse effect (Becker, 2007; Chacón-Lee & González-Mariño, 2010; Enzing et al., 2014).

One of the most important microalgae advantages is the lack of soil requirement for growing and assimilation of molecules present in aqueous environments; however, variables such as CO_2_, light, ventilation, nitrogen, phosphorus, salts, metals, and their interactions, may cause a very noticeable diversification in the type of molecules of interest and biomass produced (Kasting & Siefert, 2002).

Biologically active compounds production by microalgae has demonstrated to be nutritionally more efficient, compared with those present in traditional crops. In addition, microalgae accumulate different metabolites of biotechnology application (Bleakley & Hayes (2017). Among other microalgae, *Chlorella* species have been industrially produced based on their fast growth rates and biomass production, which has high lipid and protein contents, compared with other microalgae. In this regard, *Chlorella vulgaris* is widely commercialized as nutritional supplement for humans and as animal feed additives (Van der Spiegel, Noordam & Van der Fels-Klerx, 2013). Moreover, as nutritional supplements, proteins must show digestibility and essential amino acids availability. Proteins from animal sources have higher essential and digestible amino acids than plants. In this concern, most algae species lack producing most essential amino acids, or produce some of them at very low concentrations (Bleakley & Hayes (2017). In 2007, Morris et al. reported *in vivo* immune-potentiating activity of a *Chlorella* protein hydrolysate, suggesting a potential industrial use as physiologically functional food. Furthermore, Ursu et al. (2014) demonstrated up to seven essential amino acids and proteins production by *C. vulgari*. Since proteins resulted to have excellent emulsifying properties, they concluded that this microalga had potential as food complement or as techno-functional ingredient. In this regard, nutrients present in the microalgae biomass have been used to enrich animal food (Tibbetts, Milley & Lall, 2015). It is then important to evaluate the conditions relative to the production of these added-value molecules as food supplements (Guccione et al., 2014; Tibbetts, Milley & Lall, 2015), which mostly involves amino acids, with the potential to generate essential proteins. Furthermore, it is feasible to determine the metabolic activity of the lipids by quantifying acylcarnitines, related to the different fatty acids and their respective chain lengths. Amino acid profile and quantification of acylcarnitines assays are performed by tandem mass spectrometry, which has been used in clinical and veterinary areas of interest (Villarreal-Pérez et al., 2014; Rodríguez-Sánchez et al., 2015).

In the present study, amino acid profile and acylcarnitine production by *Chlorella vulgaris* and *Chlorella sorokiniana* in wastewater under light (L) and shaking (Sh) alone or in combination, was determined to evaluate their potential as animal food supplements.

## Material & Methods

### Microalgae growing conditions

Study was conducted following previous reports (Tibbetts, Milley & Lall, 2015; Wang et al., 2015), with slight modifications for small volumes. In brief, 2 L water samples were taken from ”La Encantada” stream, which crosses through the Asturias neighborhood, in the city of Saltillo, Coahuila, Mexico, and represents an irregular dump area for domestic and agricultural waste. These water samples were used as the culture medium for *Chlorella* microalgae species.

Microalgae inoculum was grown in modified BG11 culture medium (50 g/L glucose, 1.5 g/L NaNO_3_, 0.4 g/L K_2_HPO_4_ ⋅ 3H_2_O, 0.075 g/L MgSO_4_ ⋅ 7H_2_O, 0.036 g/L CaCl_2_ ⋅ 2H_2_O, 0.02 g/L Na_2_CO_3_, 0.006 g/L citric acid, 0.006 g/L C_6_H_8_O_7_ ⋅ x Fe_3_ + ⋅ NH_3_, and 0.001 Na_2_EDTA, at pH = 7.0) (Reyna-Martínez et al., 2015), under the following conditions: light at 1,380 lumens (lm) during the bioprocess (light:dark cycles of 12:12 h) and agitation at 130 rpm in a rotary shaker, during 18–21 d, until reaching an optical density (OD) of 0.5–0.6 at a wavelength of 647 nm (Smart Spec Plus Spectrophotometer; Bio-Rad Laboratories, Hercules, CA), which is an indication of exponential growth. Inoculum cell concentration was then determined in a Neubauer hematocytometer, showing a range of 5.6 × 10^7^ ± 7 × 10^2^ cells/mL. To start culture, 10 mL of exponentially growing culture suspensions were inoculated into seven 500 mL flasks containing 250 mL of wastewater from “La Encantada” stream, and incubated at 23 °C and natural day light, under the following conditions: flasks 1–3 = *C. sorokiniana* adding (1) extra light (12:12 light:dark cycles 1,380 lumens), no agitation; (2) agitation at 130 rpm and no extra light; (3) light at 1,380 lumens and agitation at 130 rpm; flasks 4–6: *C. vulgaris* under conditions similar to those described above; and flask 7 = microalgae untreated control, which consisted of wastewater without inoculum, incubated at 23 °C, natural day light, and 130 rpm agitation. Results represent three replicate determinations per treatment from three independent experiments.

### “La Encantada” stream water bioremediation after microalgae inoculation

Bioremediation potential of *C. sorokiniana* and *C. vulgaris* was evaluated. Before the experiment (time zero, T0), conductivity (mS), pH, turbidity (FTU), dissolved oxygen (ppm), organic matter (%), total nitrogen (%), chemical oxygen demand (mg/L) and biochemical oxygen demand (mg/L) of the residual water were evaluated (Eaton et al., 1995). After 13 d culture, these parameters were also determined in the inoculated water and in the untreated control, resulting in three treatments. Flasks with each treatment were incubated under light, agitation, and both conditions. pH and conductivity were digitally determined with the use of a high range Hanna HI 98130 multiparameter, whereas dissolved oxygen was determined with a HI 9146 meter, and turbidity with a HI93703C portable turbidimeter.

Before each measurement, instruments were calibrated based on the manufacturer’s specifications. Organic material content was determined by the potassium dichromate (K_2_Cr_2_O_7_) oxidation in presence of a strongly acidic medium (H_2_SO_4_), which is complemented by titration of the remaining oxidizing FeSO_4_. Result was expressed as organic materal percentage.

Total nitrogen was determined by the Kjheldahl method, where the sample was subjected to a wet digestion. The amount of components susceptible to oxidation to determine the chemical oxygen demand was carried out in the presence of K_2_Cr_2_O_7_ as an oxidizing agent. The biochemical oxygen demand to calculate oxidizable material amount by microbial activity was determined by the differential in the dissolved oxygen concentration after 5 d fermentation process incubated at 20 °C (Mohabansi, Tekade & Bawankar, 2011). For this, fermentation was carried out in triplicate to determine the differences between the treatments by comparison of means (*p* = 0.05).

### Microalgae amino acids and acylcarnitines production analysis

After 13 d of culture, amino acids and acylcarnitines type and amount present in microalgae biomass (1.5 mL), obtained at the end of the fermentation bioprocess, were evaluated by mass spectrometry (MS). Prior to analysis, each sample was subjected to freezing at −80 °C, followed by crushing with a pistil, and sonicating in a bath-type sonicator for 20 min to achieve a physical lysis. For metabolomic evaluation, samples were analyzed as previously reported by Martin-Park et al. (2017) with slight modifications, and profiles analyzed using the R software. In brief, 15 aminoacids and 31 acylcarnitines were extracted by a NeoBase non-derivatized liquid chromatograph coupled to mass spectrometry kit (LC-MS/MS; Perkin Elmer Life and Analytical Sciences, Turku, Finland), for further quantification, and determined by liquid LC-MS/MS (API 2000, ABSciex, Framingham, MA), which was coupled to a micropump and to a series 200 autosampler (Perkin Elmer, Norwalk, CT). Biomolecule concentrations were analyzed with Analyst 1.6.2 Software (ABSciex) and NeoBase database.

Excel was used to generate heat maps to determine amino acids and acylcarnitines production among treatments. Total biomass of each molecule group was converted to a total proportion of 1, transforming each molecule mass into a proportion based on the total production. For each proportion determination, each molecule specific mass was divided by the total metabolite production. The differences between proportions were represented by different colors in the heat maps. Tables with fold increase/decrease proportion values were generated to determine differences between shaking and light treatments alone or in combination, and subsequently compared with the untreated control.

## Results

### “La Encantada” stream water bioremediation

Conductivity value was significant lower in all treatments (values from 1.41 to 1.59 mS), than those recorded after inoculating microalgae and not incubated under light conditions (1.72 and 1.76 mS by *C. vulgaris* and *C. sorokiniana*, respectively), compared with time 0 (2.22 mS) (Table 1). In regard to pH, values were significantly higher in most treatments (ranging from pH from 9.04 to pH 9.42) compared with time 0 (8.35), except for *C. sorokiniana* cultured under agitation (pH = 8.8) and the untreated control (pH = 9.11) (Table 1).

**Table 1:** Water biorremediation. Physicochemical parameters of La Encantada stream water after inoculation with *Chlorella sorokiniana* and Chlorella vulgaris 13 d culture under light (L), agitation (Sh) or both (L + Sh ) conditions.

Parameter ± St error	T0	Control	*C. vulgaris*			*C. sorokiniana*		
			L + Sh	L	Sh	L + Sh	L	Sh
Cond (mS)	2.22 ± 0.31a	1.59 ± 0.22b	1.48 ± 0.21b	1.41 ± 0.19b	1.72 ± 0.26ab	1.5 ± 0.1b	1.41 ± 0.18b	1.76 ± 0.19ab
pH	8.35 ± 0.15b	9.11 ± 0.74ab	9.38 ± 0.35a	9.42 ± 0.4a	9.04 ± 0.44a	9.4 ± 0.8a	9.35 ± 0.54a	8.8 ± 0.37b
Turbidity (FTU)	115 ± 15a	9.84 ± 1.52c	17.64 ± 2.1b	2.62 ± 0.39e	21.0 ± 4.25b	7.7 ± 1.4c	3.07 ± 0.15e	4.64 ± 0.5d
DO (ppm)	2.84 ± 0.07b	7.54 ± 1.66a	6.65 ± 1.65a	6.72 ± 1.47a	7.75 ± 1.87a	7.3 ± 2.3a	6.89 ± 1.92a	6.36 ± 0.41a
OM total (mg/L)	103 ± 32a	104 ± 30a	48 ± 1.0b	101 ± 29a	106 ± 13a	50 ± 20b	102 ± 18a	40 ± 5.0a
N total (mg/L)	20 ± 8.0a	23 ± 7.0a	14 ± 8.0ab	21 ± 6.0a	18 ± 7.0ab	10 ± 0.0b	20 ± 3.0a	10 ± 3b
COD (mg/L)	335 ± 8.7a	406.7 ± 96a	381.7 ± 44a	152.3 ± 6.8c	91.3 ± 20.1d	216.7 ± 30b	196.7 ± 40b	85 ± 21.8d
BOD5 (mg/L)	48.8 ± 16.6a	37.7 ± 0.96a	44.97 ± 8.0a	36.53 ± 5.9a	21.13 ± 5.3b	33.2 ± 6.2ab	28.03 ± 10ab	28.3 ± 2.5b

**Notes.**

St error standard error T0 time zero Cond conductivity mS milisiemens FTU formazine turbidity unit DO dissolved oxygen ppm parts per million OM organic material N nitrogen COD chemical oxygen demand BOD5 biochemical oxygen demand after 5 d fermentation mg/L miligrams/liter

Average of three replicates.

Diferent letters after the value in the same row represent significant differences (*p* = 0.05) by minimum differences of means.

Turbidity was significantly higher at time 0 (115 FTU) compared with all other treatments, followed by *C. vulgaris* cultured under agitation with or without light (17 and 21 FTU, respectively), untreated control (9.8 FTU) and *C. sorokiniana* cultured under agitation and light (7.7 FTU), and *C. sorokiniana* cultured under agitation (4.6 FTU). Significantly low turbidity values were detected in *C. vulgaris* and *C. sorokiniana* cultures under light conditions (2.6 and 3.0 FTU, respectively) (Table 1). Results of the dissolved oxygen demonstrated that all treatments were significantly high, ranging from 6.36 to 7.54 ppm, compared with time 0 (2.84 ppm) (Table 1).

In contrast, organic matter was significantly higher in all treatments, ranging from 101 to 104 mg/L, compared with that of *C. vulgaris* and *C. sorokiniana* cultured under light and agitation conditions (48 and 50 mg/L, respectively).

Total nitrogen in *C. vulgaris* cultures under agitation with or without light (14 and 18 mg/L, respectively), were not different compared with all other treatments, whereas at time 0, untreated control, *C. vulgaris,* and *C. sorokiniana* cultured under light conditions resulted in values significantly higher, ranging from 20 to 23 mg/L, compared with those in *C. sorokiniana* cultures under agitation conditions with or without light (10 mg/L by both treatments) (Table 1).

Chemical oxygen demand resulted in significantly higher values at time 0, untreated control, and *C. vulgaris* cultured under agitation and light conditions compared with all other treatments, ranging from 335 to 406 mg/L, followed by *C. sorokiniana* cultured under light condition (197 mg/L), and *C. vulgaris* and *C. sorokiniana* cultured under agitation (no-light condition) (91 and 85 mg/L, respectively) (Table 1).

Biochemical oxygen demand in *C. sorokiniana* cultured under light with or without agitation (33 and 28 mg/L, respectively), was not different compared with all other treatments, whereas at time 0, untreated control and *C. vulgaris* cultures under light with or without agitation conditions resulted in significantly higher values, ranging from 36.5 to 48.8 mg/L, compared with those of *C. vulgaris* and *C. sorokiniana* cultured under agitation (21 and 28 mg/L, respectively) (Table 1).

### Microalgae amino acids and acylcarnitines production

A fold-type analysis was used to express the number of times that the proportions of a combined treatment increase or decrease, as compared with treatments alone. In the case of *C. sorokiniana* acylcarnitine C0, when comparing the production under light conditions against both conditions, a proportion of 0.84 was observed, corresponding to a smaller amount of the molecule detected, similarly as when comparing shaking and both treatments (0.95) (Table 2). When analyzing the case of C4, under light conditions there was a higher value (1.55) than that in both treatments, contrary to that showed with shaking, where the proportion was lower (0.65) than the use of the combined treatment. After comparing C0 production by the untreated control, an increase in C0 production was observed (1.87, under combined conditions; 2.23, light); which was not observed after analyzing C4 production under light exposure (−0.8321) (Table 2).

After analyzing the *C. sorokiniana* culture amino acids quantity, higher production of glycine (Gly), alanine (Ala), and leucine (Leu) was highlighted. The fold values indicated that Gly had a higher production under shaking with a ratio of 1.05, as compared with light and shaking applied at the same time in contrast to light treatment, where production was lower, in relation to both treatments (0.78) (Table 3). In regard to Ala production, the application of light and combining light and agitation produced a similar amount of amino acids, both higher than the amount produced by agitation alone. Furthermore, the production of Leu under light and agitation conditions applied at the same time, was lower than that produced in only one condition (light = 1.10; agitation = 1.04). Production results by the untreated control revealed a higher Gly amount compared with that of treatments using combined conditions and microalgae under light (0.234 and 2.7463, respectively). In contrast, Ala production was low in microalgae biomass (−0.5252 and −0.6428, for the combined conditions and agitation, respectively). Leu production was high in microalgae biomass under agitation (0.1344), but it was present in low amounts when microalgae were cultured under both light and agitation conditions (−0.1396), compared with that of untreated control.

**Table 2:** Acylcarnitines production variation by *Chlorella sorokiniana* under different culture conditions.

**Metabolite**	**Fold change (light vs light + shaking)**	**Fold change (shaking vs light + shaking)**
**C0**	−0.8388	−0.9473
**C4**	+1.5493	−0.6516

**Notes.**

− decrease production + increase production

For *C. vulgaris*, C0 was produced in higher proportion when light and agitation were separately applied (light = 1.27; agitation = 1.7), as compared with the combined culture conditions. In contrast, C6DC production was higher in the combined culture conditions, as compared with only one condition (light = 0.72; agitation = 0.82). After comparing these results with the untreated control, a lower C0 production was observed related to culture condition with agitation and combination of light and agitation (−0.736 and −0.4319, respectively). Light and agitation culture condition resulted in a higher C6DC amount (−0.9464), compared with that of untreated control, but was similar when microalgae were cultured combining light and agitation (0.7794) (Table 4).

**Table 3:** Amino acids production variation by *Chlorella sorokiniana* under different culture conditions.

**Amino acid**	**Fold change (light vs light + shaking)**	**Fold change (shaking vs light + shaking)**
Glycine	−0.7826	+1.0507
Alanine	1.0000	−0.8171
Leucine	+1.1005	+1.0391

**Notes.**

− decrease production + increase production

A different phenomenon was observed when analyzing C4, where there was a higher production when *C. vulgaris* only grew under light condition (1.07), as compared with combined treatments, in contrast to shaking alone whose C4 production was lower than the combined treatments (0.82). Conversely, C4 production in untreated control was higher than that produced by microalga under light and agitation conditions (2.03 and 2.46, respectively) (Table 4).

After quantifying Leu and Val, a higher percentage of these amino acids was observed when *C. vulgaris* was cultivated under both conditions at the same time, compared with light (Leu = 0.91 and Val = 0.97) and shaking (Leu = 0.86 and Val = 0.85) conditions alone (Table 5). Ala showed a higher proportion when the strain was grown under light and shaking separately, than when both conditions were applied at the same time; higher production of Gly was observed when growing this strain under light (1.23), in contrast to growing it under shaking (0.89), as compared with amino acids production under both conditions (Table 5). Leu (−0.1231 and −0.1436), Ala (−0.5625 and −0.54) and Val (−0.4695 and −0.551) production were lower in untreated control, as compared with those in microalgae cultured either under agitation alone or agitation and light; in contrast, Gly production was higher in untreated control (4.16 and 4.68, respectively).

**Table 4:** Acylcarnitines production variation by *Chlorella vulgaris* under different culture conditions.

**Metabolite**	**Fold change (light vs light + shaking)**	**Fold change (shaking vs light + shaking)**
C0	+1.268	+1.704
C6DC	−0.7205	−0.8235
C4	+1.0735	−0.8235

**Notes.**

− decrease production + increase production

**Table 5:** Amino acids production variation by *Chlorella vulgaris* under different culture conditions.

**Amino acid**	**Fold change (light vs light + shaking)**	**Fold change (shaking vs light + shaking)**
Leucine	−0.9113	−0.8571
Alanine	+1.0708	+1.0416
Glycine	+1.2307	−0.8901
Valine	−0.9739	−0.8521

**Notes.**

− decrease production + increase production

In regard to the heat maps, the colors closest to a red tone showed the values that were produced in higher amounts, whereas those closest to a yellow color had lower production; complementing the above and expressed in proportions, the totality of acylcarnitines provided a total sum of 1, therefore each number expressed in the Figure indicated the proportion in which each molecule was produced.

**Figure 1:**
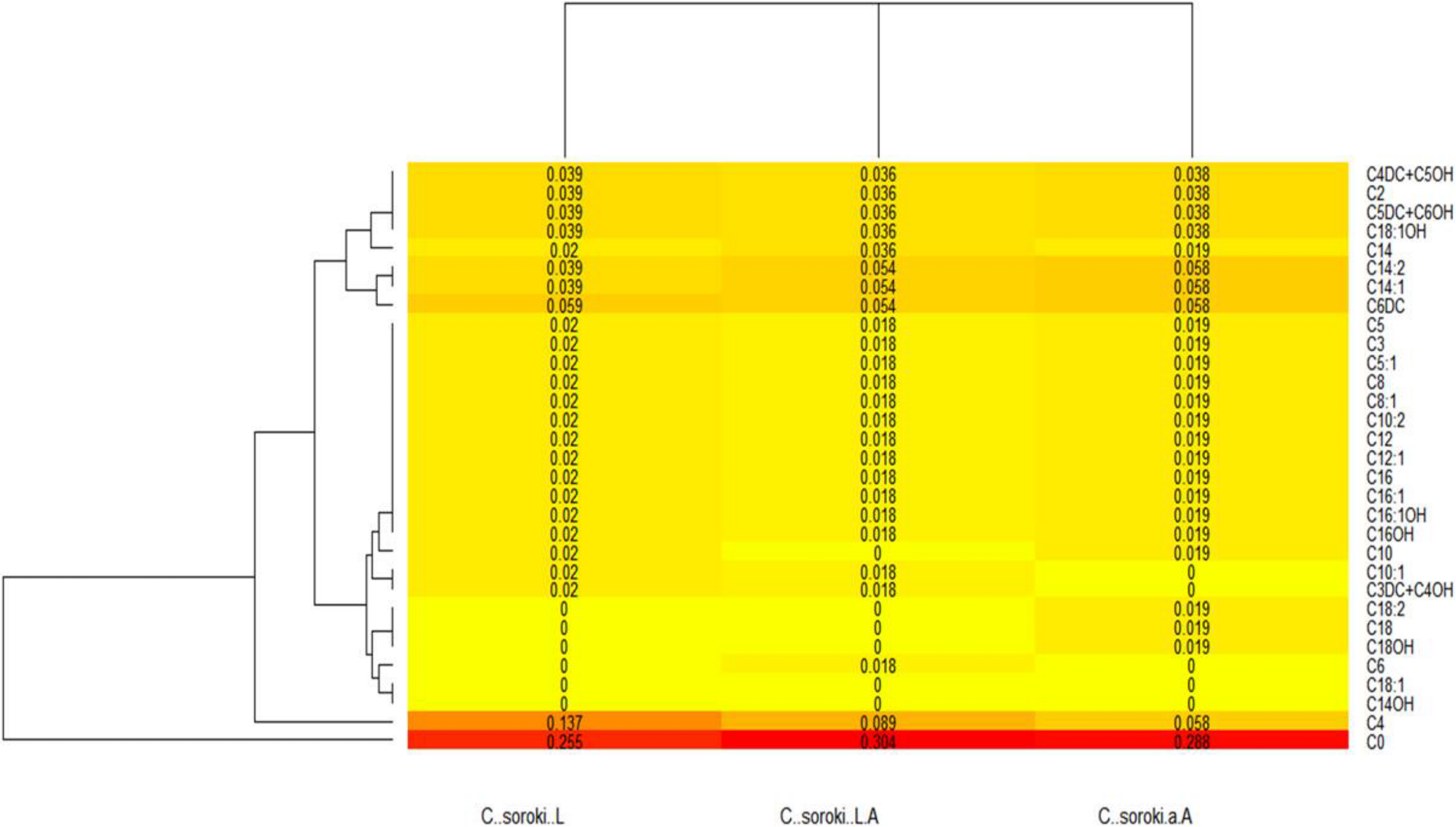
*Chlorella sorokiana*’s acylcarnitines profiles heatmap. Variation in the production of acilcarnitines, under all the culture conditionsis shown. C..soroki.L = under light culture, C..soroki.A = under shaking culture, and C..soroki.L.A = under light + shaking culture.

**Figure 2:**
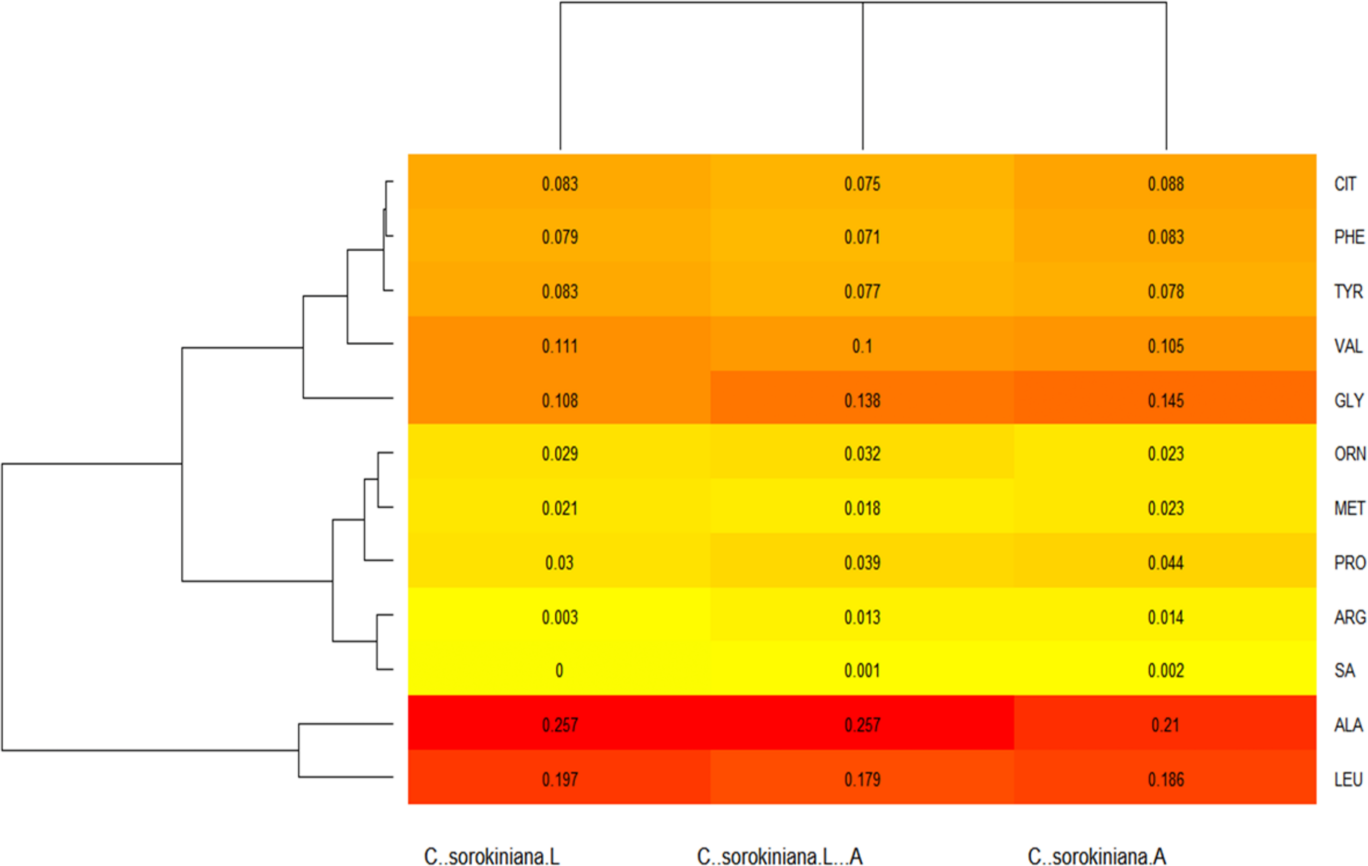
*Chlorella sorokiana*’s amino acids (AA) profiles heatmap. Variation in the production of amino acids, under all the culture conditions (light, shaking and both) is shown. C..sorokiniana.L = under light culture, C..sorokiniana.A = under shaking culture, and C..sorokiniana.L..A = under light + shaking culture. ALA, alanine; ARG, arginine; CIT, citrulline; GLY, glycine; LEU, leucine; MET, methionine; ORN, ornithine; PHE, phenylalanine; PRO, proline; SA, serine; TYR, tyrosine; VAL, valine.

In the heat map, the ratio of acylcarnitines produced under the different *C. sorokiniana* culture conditions was shown in Fig. 1. C0 was produced in large amounts under the three culture conditions, followed by acylcarnitine C4, which was synthesized in higher amounts when the microalga was only grown under the influence of light, but it decreased under both conditions. In addition, the map showed the absence of the acylcarnitines C14 OH and C18: 1 under the three culture conditions. In the case of C18, C18 OH, and C18: 2, they were observed when the strain was grown only under shaking. C6 was only produced under light and shaking conditions and all those mentioned above were not produced when the strain grew under lighting. Microalgal C14:1 and C14:2 productions were lower compared with that produced by untreated control. Observing the heat map (Fig. 2), the amino acids production by *C. sorokiniana* was directed towards some of the essential amino acids group.

Ala production was mostly observed under light conditions and both conditions, with a lower production observed under shaking. In the case of Leu, it was produced in higher amounts under light, as compared with combined treatments. Similarly, Leu was produced only under shaking conditions, as compared with untreated control. Furthermore, Gly was produced in higher amounts under both conditions at the same time or shaking alone, as compared with light condition; however, microalgae cultures resulted in lower production compared with untreated control. Val is produced by a similar pathway under the three conditions, as similarly observed by tyrosine (Tyr). Under both conditions, phenylalanine (Phe) was produced in less quantity, as compared with light and shaking alone, and in all three cases, negative control produced lower amount of metabolites.

**Figure 3:**
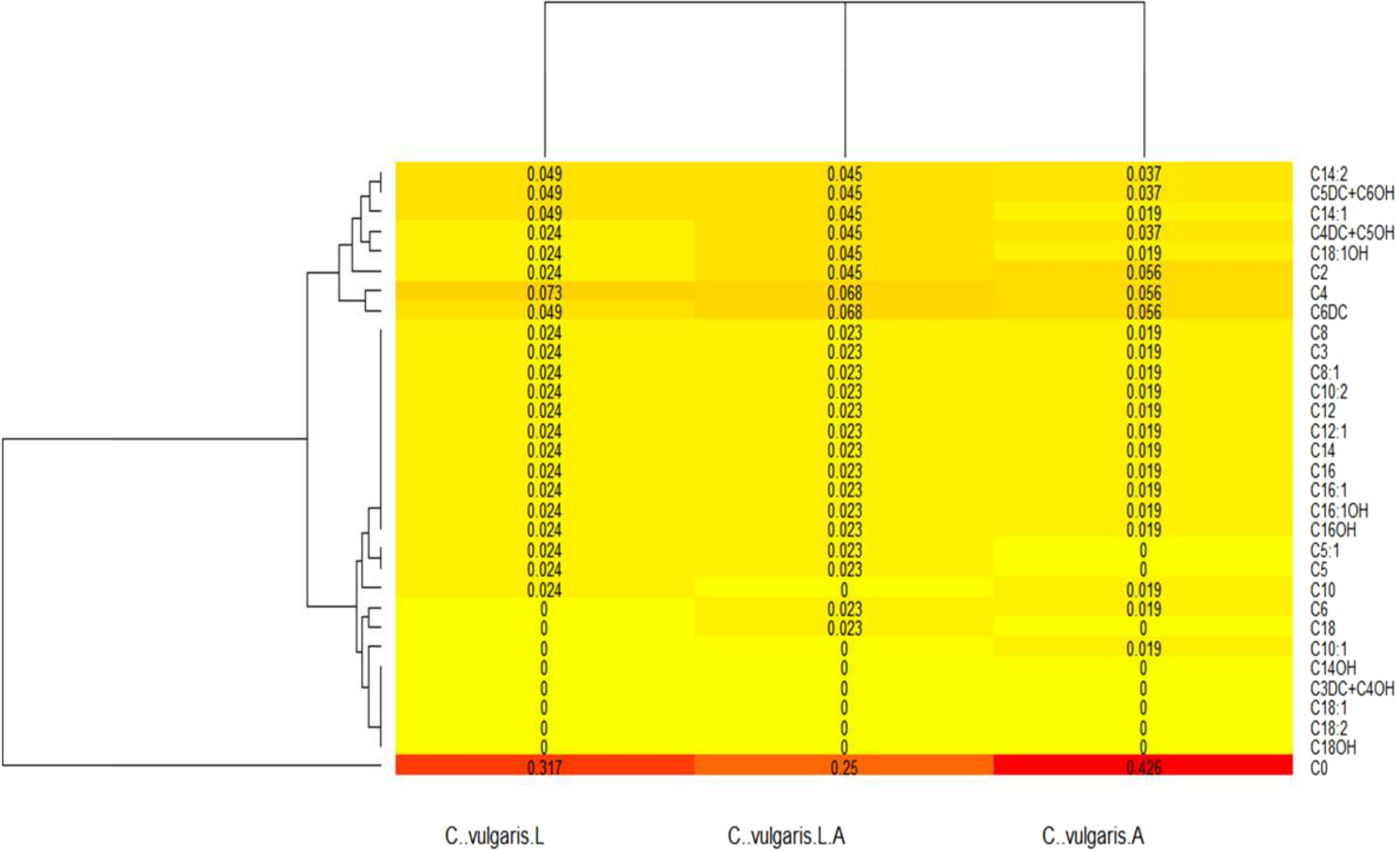
*Chlorella vulgaris*’s acylcarnitines profiles heatmap. Variation in the production of acilcarnitines, under all the culture conditions (light, shaking and both) is shown. C..vulgaris.L = under light culture, C..vulgaris.A = under shaking culture, and C..vulgaris.L..A = under light + shaking culture.

By analyzing the heat map of the production of acylcarnitines in *C. vulgaris*, under the different culture conditions (Fig. 3), the production of C0 under the three conditions was observed, having higher production when the strain was grown under shaking, following in order by light and combined treatments; in addition, CO production by untreated control was lower compared with that of microalgae under any culture conditions. The map also showed an absence in the production of C14OH, C3DC + C4OH, C18: 1, C18: 2, and C18OH under the three growing conditions, whereas a small amount of these metabolites were produced by the untreated control. However, heat maps showed no differences between treatments.

**Figure 4:**
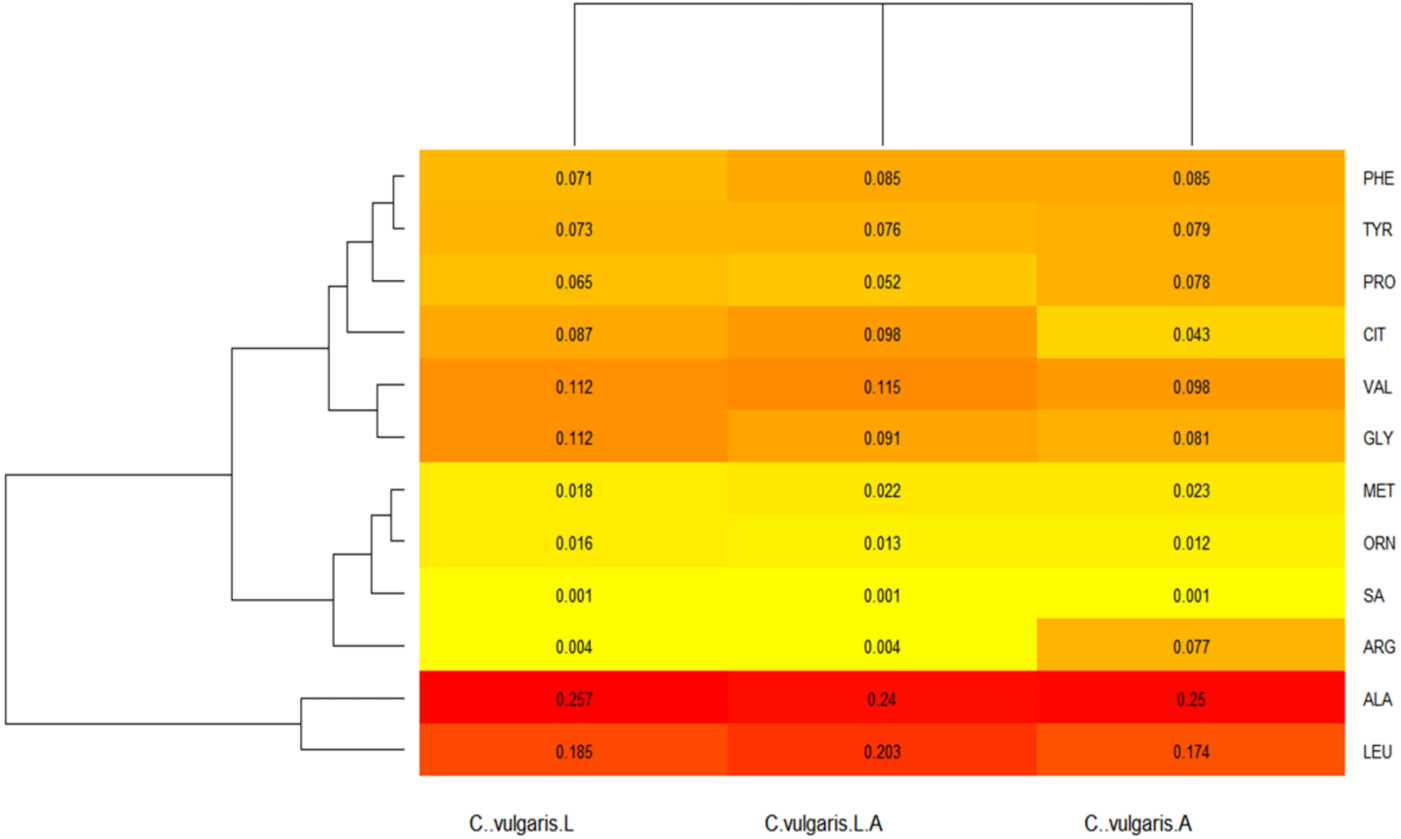
*Chlorella vulgaris*’s amino acids (AA) profiles heatmap. Variation in the production of amino acids, under all the culture conditions (light, shaking and both) is shown. C..vulgaris.L = under light culture, C..vulgaris. A = under shaking culture, and C..vulgaris.L..A = under light + shaking culture. ALA, alanine; ARG, arginine; CIT, citrulline; GLY, glycine; LEU, leucine; MET, methionine; ORN, ornithine; PHE, phenylalanine; PRO, proline; SA, serine; TYR, tyrosine; VAL, valine.

*C. vulgaris* amino acids production in the heat map yielded the data depicted in Fig. 4. Ala was produced in significant amounts, under the three growing conditions, as compared with untreated control. Leu was produced in higher amounts when microalgae were cultured under both conditions, as compared with light and agitation alone; however, Leu was produced by the untreated control as well.

A similar production of Gly and Val was observed under light conditions, a phenomenon also observed in the production of Val when the strain was cultivated under both conditions at the same time. In contrast to untreated control, Val production by microalgae under those culture conditions was higher compared with that of untreated control, contrary to what was observed by Gly production, where the control showed higher production. Furthermore, production of phenilalanine (Phe), proline (Pro), and tyrosine (Tyr), were detected under agitation conditions. Tyr resulted in high production after microalgae were cultured under light alone or combined with agitation, whereas citruline (Cit), Phe, Pro, and Tyr production was higher in cultures under light conditions. In addition, Cit, Phe, and Tyr were produced in higher amounts by microalgae, as compared with those in untreated control, contrary to that observed by Pro production (Fig. 4).

## Discussion

Bioremediation of stream water demonstrated that conductivity was under the standard limit value (8 mS) (NOM-CCA-032-ECOL/1993, http://paot.org.mx/centro/normas/031-ecol.pdf, accessed by Aug-24-2019); whereas the pH value at time zero (pH = 8.35) was the only value under the standard limit (pH = 8.5) by the Mexican norm (NOM-001-SEMARNAT/1996, https://www.profepa.gob.mx/innovaportal/file/3290/1/nom-001-semarnat-1996.pdf, accessed by Aug-24-2019).

Turbidity values were above the standard limit (5.0 FTU) but *C. sorokiniana* cultured under agitation (4.6 FTU), and *C. vulgaris* and *C. sorokiniana* cultured under light conditions (2.6 and 3.0 FTU, respectively) (NOM-127-SSA-1-1994, modified by 2000) (https://agua.org.mx/wp-content/uploads/2016/10/nom127_modificacion_2000.pdf, accessed by Aug-24-2019). The Mexican norm does not establish a limiting value of dissolved oxygen in water.

Analysis of total nitrogen revealed that all treatments were under the standard limit (40 mg/L) (NOM-001-SEMARNAT/1996). The only treatments that showed values under the standard limit by chemical oxygen demand (210 mg/L) were *C. sorokiniana* cultured under light condition (197 mg/L), and by *C. vulgaris* and *C. sorokiniana* cultured under agitation no-light condition (91 and 85 mg/L, respectively) (NOM-001-SEMARNAT/1996). In contrast, biochemical oxygen demand values demonstrated that all treatments were under the standard limit (70 mg/L) (NMX-AA-028, NOM-001-SEMARNAT/1996).

Metabolites analysis by MS is commonly performed in clinical samples to determine metabolic disorders, toxicology, drug trafficking, metabolic genetics, and analysis of acylcarnitines linked to lipolytic pathways (Chace, 2009; Afshinnia et al., 2018). The present study produced a novel perspective of the intracellular metabolites that are related to the metabolism of proteins and lipids, since in the reviewed literature there were no reports of these studies in environmental samples, mainly referring to microalgae. Analyzing the amino acids proportions detected, there were some amino acids belonging to the essentials group, similar to that reported by FAO/WHO (1971) showing the presence of Val, Pro, Leu, Phe, Tyr, Ala, and Gly.

Microalgae consumption as food supplement has not proven to be toxic when used as a food or supplement (Draaisma et al., 2013), therefore it becomes a nutritional option due to the content of essential amino acids, which have the characteristic of increase the expression of genes related to the metabolism of proteins, for example, in muscle recovery (Børsheim et al., 2002). The pattern of amino acid production in *C. sorokiniana* was presented in a similar way as in *C. vulgaris*, despite the fact that studies on *C. sorokiniana* have been mainly focused to the production of lipids used as biofuels (Lu et al., 2012); this strain also has potential use as a food supplement with similar effects on protein metabolism. Within lipids metabolism, medium and long chain fatty acids, mainly for their oxidation and introduction to the mitochondria, must be activated by the formation of acylcarnitine’ complexes, which will be introduced to the mitochondrial matrix by means of acylcarnitine complex transfers (Yu et al., 2018). In clinical studies, evidence has been presented of the relationship between the acylcarnitine profile and the diagnosis of metabolic diseases (Roe et al., 1985). There are no reports showing microalgae acylcarnitine profiles, this directed the investigation towards an analysis of the metabolites present in the samples under the culture conditions described above, focusing on the type of fatty acids that, in theory, are being metabolized in the cell.

As previously reported by Hu et al. (2008), most Chlorophyta fatty acids are C16: 0- C18: 1; similarly, in the present study it was observed that most acylcarnitines were conjugated with medium chain fatty acids in a range of C10–C18 in both strains, suggesting that these fatty acids are introduced into mitochondria for oxidation. This is supported by the high proportion of C0 (free carnitine), related to the beta oxidation process; in addition to this and in comparison with the higher plants, it has been determined that the accumulation of these components in the microalgae is low, since they lack tissues present in plants (Petkov & Garcia, 2007). The proportion of acylcarnitine C3, malonylcarnitine in the metabolomic analysis of both strains, follows a low induction of fatty acid biosynthesis, a process carried out in the microalgae endoplasmic reticulum (Bellou et al., 2014).

Production of molecules related to the proteins and lipids metabolism in both strains relies on light and shaking conditions. A higher production of Leu, Ala, Val, and Gly, and lower production of Pro, Tyr, and Phe essential amino acids were observed in both microalga strains. Acylcarnitines present in *C. sorokiniana* cultured under the three conditions, were mostly bound to medium-chain fatty acids (C5–C18), regardless if the microalga was produced in low amounts; however, high levels of C0, defined as free carnitine and C4 (succinyl carnitine) were observed without being conjugated with fatty acyl (C0) or potentially being a precursor of succinyl Co A (C4), which interacts with the Krebs cycle in the amino acids metabolism. *C. vulgaris* culture showed a similar acylcarnitine types production; however, there was a lower proportion of C4, compared with that of *C. sorokiniana*, thus suggesting a slightly lower production of the Krebs cycle precursors and consequently, lower protein metabolism (Petkov & Garcia, 2007). In addition, the acylcarnitine C3 (malonylcarnitine), important in the polyunsaturated fatty acids and triglycerides biosynthesis in the endoplasmic reticulum of plant cells, was detected under nutritional proportion levels (Liu & Hu, 2013; Koller, Muhr & Braunegg, 2014).

## Conclusions

Evaluated microalgae produced essential amino acids and nutritionally important carnitines; however, *C. sorokiniana* has significant potential as animal nutrient supplement.

It is important to highlight that the metabolomic and MS analysis performed in this study were useful to determine the microalgae nutritional potential, as a complementary tool to understand the possible metabolic state of the cell, recognizing that the combination between culture conditions and identified molecules can provide data that may support optimizing culture media, which may stimulate production of metabolites of interest by the biotechnology industry.

##  Supplemental Information

10.7717/peerj.7977/supp-1 Supplemental Information 1 Wastewater analysis Water biorremediation by treatments

10.7717/peerj.7977/supp-2 Supplemental Information 2 Experiment Report

## Additional Information and Declarations

### Data Availability

The following information was supplied regarding data availability: Raw data is available in the Supplemental Files.
